# Predicting the Timing of Cherry Blossoms in Washington, DC and Mid-Atlantic States in Response to Climate Change

**DOI:** 10.1371/journal.pone.0027439

**Published:** 2011-11-07

**Authors:** Uran Chung, Liz Mack, Jin I. Yun, Soo-Hyung Kim

**Affiliations:** 1 Center for Urban Horticulture, School of Forest Resources, College of the Environment, University of Washington, Seattle, Washington, United States of America; 2 Department of Ecosystem Engineering, Kyung Hee University, Yongin, Korea; 3 National Center for Agro-Meteorology, Seoul National University, Seoul, Korea; Netherlands Institute of Ecology, The Netherlands

## Abstract

Cherry blossoms, an icon of spring, are celebrated in many cultures of the temperate region. For its sensitivity to winter and early spring temperatures, the timing of cherry blossoms is an ideal indicator of the impacts of climate change on tree phenology. Here, we applied a process-based phenology model for temperate deciduous trees to predict peak bloom dates (PBD) of flowering cherry trees (*Prunus×yedoensis* ‘Yoshino’ and *Prunus serrulata* ‘Kwanzan’) in the Tidal Basin, Washington, DC and the surrounding Mid-Atlantic States in response to climate change. We parameterized the model with observed PBD data from 1991 to 2010. The calibrated model was tested against independent datasets of the past PBD data from 1951 to 1970 in the Tidal Basin and more recent PBD data from other locations (e.g., Seattle, WA). The model performance against these independent data was satisfactory (Yoshino: *r^2^* = 0.57, RMSE = 6.6 days, bias = 0.9 days and Kwanzan: *r^2^* = 0.76, RMSE = 5.5 days, bias = −2.0 days). We then applied the model to forecast future PBD for the region using downscaled climate projections based on IPCC's A1B and A2 emissions scenarios. Our results indicate that PBD at the Tidal Basin are likely to be accelerated by an average of five days by 2050 s and 10 days by 2080 s for these cultivars under a mid-range (A1B) emissions scenario projected by ECHAM5 general circulation model. The acceleration is likely to be much greater (13 days for 2050 s and 29 days for 2080s ) under a higher (A2) emissions scenario projected by CGCM2 general circulation model. Our results demonstrate the potential impacts of climate change on the timing of cherry blossoms and illustrate the utility of a simple process-based phenology model for developing adaptation strategies to climate change in horticulture, conservation planning, restoration and other related disciplines.

## Introduction

Warming associated with climate change has been shown to alter ecosystem processes including phenology – the timing of organism development [1], [2]. The phenology of plants is sensitive to changes in temperature. During the past decades, considerable shifts in tree phenology have been reported in the temperate regions; these shifts are likely to be a response to the changing climate. For example, Mynei et al. [3] and Parmesan and Yohe [4] demonstrated that the growing season of trees has increased by 2.3 days in the past 40 years. Richardson et al. [5] reported a similar increase of 2.1 days in temperate tree species. In Washington, DC area, 89 of 100 plant species surveyed, including flowering cherry trees, exhibited a significant advance of 4.5 days in first-flowering over the 30 years from 1970 to 1999 [6]. It has been predicted that these trends will continue into the 21st century [7]. The expected changes in phenology will have a substantial effect on the reproduction, distribution and productivity of trees as the coincidence of ecosystem processes, such as flowering and the emergence of pollinators, is disrupted [8]. Some plants may also become less resistant to environmental challenges. For example, shorter and warmer winters can reduce the cold hardening of trees, leaving them vulnerable to frost injury [9].

Simple, thermal-time based phenology models can provide useful insights for predicting the effects of temperature on the phenology of plants, particularly in those species (e.g., cherries) that are weakly sensitive or insensitive to precipitation [6] and to photoperiod [10], [11]. When tested against the historical phenology records of leaf fall, budburst, and flowering, such models can be powerful tools to forecast the impacts of climate change on phenology, and to help develop effective adaptation strategies in agriculture, horticulture, forestry, conservation planning, restoration, and natural resource management.

Flowering cherry trees are an effective indicator of the impact of climate change on phenology because their flowering time is highly sensitive to temperatures, especially during winter and early spring (i.e., February and March) [12]. From a cultural viewpoint, an accurate prediction of cherry phenology is critical because many spring festivals and events around the world are timed around a specific phenological event – peak bloom dates (PBD). For example, the blossom of flowering cherry species (e.g., *Prunus serrulata, Prunus*×*yedoensis,* and *Prunus subhirtella*) is celebrated with festivities in many parts of the world including the U.S., Europe, and Asia (i.e., Washington, DC, USA; Tokyo and Kyoto, Japan; Jinhae and Seoul, Korea). Historically, the National Cherry Blossom Festival in Washington, DC has been taking place during the first two weeks in April. However, in the last few decades the cherry trees have been blooming earlier causing a mismatch between peak blooms and the parade that highlights the end of festival (Project BudBurst, http://www.neoninc.org/budburst/index.php, accessed 6/21/2011).

These cherry blossom festivals of spring are culturally and economically important events, and successful planning requires that the cherry blossoms appear as expected within the festival period. In Japanese culture, cherry blossoms carry great spiritual significance and their blooming has been celebrated with rituals called *hanami* since the 9^th^ century [13]. In Washington, DC, the National Cherry Blossom Festival commemorates a 1912 gift of 3020 trees from the Mayor Yukio Ozaki of Tokyo as a symbol of the friendship between the United States and Japan [14]. For these reasons, the timing of cherry blossom engenders strong public interest and cultural attentions worldwide. Furthermore, the cultural and economic significance of the flowering cherries has yielded a series of rich, long-term, phenological data sets in many cultures enabling scientists to study tree responses to climate change [15], [16], [17]. In a rapidly changing climate, predicting the flowering dates based solely on past history is likely to become less reliable; hence a more robust predictive model is needed not only for planning purposes of these cultural events but also, perhaps more importantly, for assessing the agricultural and ecological impacts of climate change.

Previously, several studies [16], [18], [19] have shown that a thermal-time based two-step phenology model successfully predicted flowering time of temperate tree species including fruit crops and flowering cherries such as *Prunus serrulata* var. *spontanea*. Chung et al. [16] extended the model for the indigenous flowering cherries in Korea and applied it to predict current and future flowering dates in South Korea. Chung et al. [16] concluded that under the A2 emission scenario, the peak bloom dates of South Korean cherry trees is likely to occur much earlier by an average of 21 days by 2050. Regionally, the spatial variability of the predicted blooming dates increased in the late 21^st^ century, but overall it has been predicted that the peak blooms are likely to take place on average 29 days earlier by 2080 [16].

Despite over 60 years of peak bloom data, no study to our knowledge has attempted to forecast the future bloom dates of the cherry trees at the Tidal Basin of Washington, DC. These cherry trees were propagated in 1911 from scions from 12 selections from the Ekita-mura area of Japan and planted in the spring of 1912 [14]. The Yoshino (*Prunus*×*yedoensis* ‘Yoshino’) cherry is the most abundant cultivar in the Tidal Basin. It is a hybrid of unknown origin from Japan with a significant historical, cultural, and economic importance for the region [20]. Significant genetic similarities have been reported between *P.*×*yedoensis* accessions in the Tidal Basin and *P. serrulata* var. *spontanea* from Korea [21]. The Kwanzan (*Prunus serrulata* ‘Kwanzan’), a double flowering pink cherry, is another abundant cultivar with 44 trees at the Tidal Basin. These two varieties are also widely cultivated throughout the U.S.

In the present study, we have modified the thermal-time based phenology model developed by Cesarracio et al. [18] and applied it to predict PBD of Yoshino and Kwanzan cherries in the Tidal Basin. We chose to use this model because 1) it has been successfully used with cherries and other temperate tree species that are highly sensitive to winter and spring temperatures in other regions [16], [18], [19], 2) it is a relatively simple but robust, process-oriented model based on physiological knowledge, and 3) it requires minimal input data (i.e., daily maximum and minimum temperatures). The objectives of the study were to 1) test the model performance for predicting PBD of the two cherry cultivars at the Tidal Basin, Washington, DC against historical records and 2) apply the model to forecast future cherry PBD in Washington, DC and the surrounding Mid-Atlantic region based on future climate projections with A1B or A2 emission scenario [7]. Briefly, the A1B scenario represents a future world of rapid economic growth, global population that peaks in mid-century and declines thereafter, and rapid introduction of novel and efficient technologies that are balanced across various energy sources. The A2 scenario assumes a very heterogeneous world with continuously increasing global population and regionally oriented economic growth that is more fragmented and slower than in other storylines [7].

## Methods

### Model description

**Figure 1:**
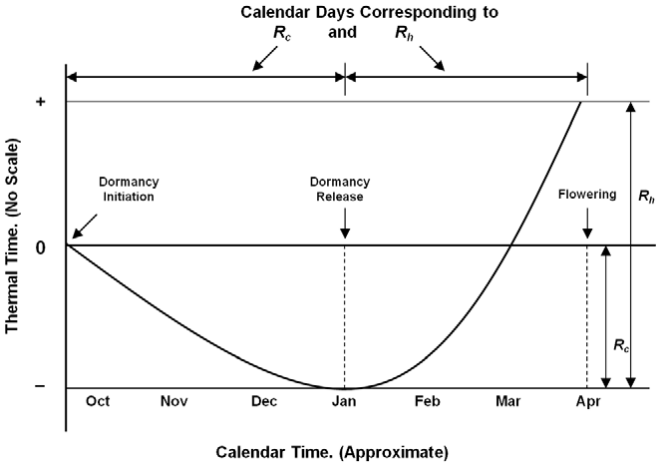
Conceptual model for predicting flowering date in temperate zone deciduous trees. Floral buds must be exposed sequentially to long enough periods of chilling temperature (*R_c_*) and heating temperature (*R_h_*) for spring flowering.

We estimated peak bloom dates (PBD) of the flowering cherry trees (*Prunus*×*yedoensis* ‘Yoshino’ and *Prunus serrulata* ‘Kwanzan’) in the Tidal Basin of Washington, DC based on the model developed by Cesaraccio et al. [18]. The bud-burst model by Cesaraccio et al. [18] was extended to predict PBD as described in Jung et al. [19] and Chung et al. [16]. Briefly, the model used in the present study divides the flowering process of deciduous trees into two stages: dormancy including rest and quiescent periods during autumn and winter, and the flowering period following bud-burst in spring (Fig. 1). The model requires the date in autumn when temperature falls below a threshold temperature, causing floral buds to enter the rest period of dormancy, and an estimation of three parameters. The threshold temperature (*T*_c_) is the base temperature below which the chill days (*D*_c_) are accumulated daily since the onset of dormancy until the chilling requirement (*R*_c_) is met. If *R*_c_ is satisfied, rest (endodormancy) is released and the heat (or anti-chill) days (*D*_h_) begins to accrue towards the heating requirement (*R*_h_). The peak bloom date is determined when *R*_h_ has been satisfied past the bud-burst. The rate of *D*_c_ and *D*_h_ accumulation depends on the daily air temperatures – mean (*T*_a_), maximum (*T*_max_), and minimum (*T*_min_) – relative to species specific temperature thresholds as detailed in Cesaraccio et al. [18] and Jung et al. [19]. In our approach, the rate of endodormancy release is tracked by the accumulation of *D*_c_/*R*_c_ towards unity at which point the resting period is over. Similarly, the rate of floral development after the resting period is modeled by daily accumulation of *D*_h_/*R*_h_.

The onset of dormancy in deciduous trees can be approximated by the date at which the temperature falls below a fixed threshold, the date that fruits are harvested from trees, or the date when the leaves begin to fall [22]. Similarly, it has been proposed that floral buds of deciduous trees enter dormancy in the autumn as their leaves begin to fall [23], [24], [25]. However, with the lack of data for and a standard method to estimate growth cessation, leaf fall, and dormancy induction period, we assumed that the onset of dormancy begins on October 1 in the present study. A similar approach has been used in other phenology modeling studies [e.g.,26,27,28].

### Model parameterization

We applied the model described above using the daily air temperature data from 1951 to 2010 collected by a weather station located near the Reagan National Airport (4.13 km from the Tidal Basin) in Washington, DC to predict PBD of Yoshino and Kwanzan cherries. The PBD of the Yoshino and the Kwanzan cherry trees in the Tidal Basin from 1921 to 2010 were obtained from Mr. Robert DeFeo, chief horticulturalist at the National Park Service (NPS). In this data set, PBD are defined as the days in which 70 percent of the blossoms of cherry trees that surround the Tidal Basin are open (pers. comm., Robert DeFeo). We performed an optimization process to derive a new set of parameter estimates for Yoshino and Kwanzan cherry trees in the Tidal Basin using the daily weather data from the Reagan National Airport and PBD data between 1991 and 2010. We used SAS NLIN procedure (SAS Institute, Cary, NC) for parameter optimization in combination with a range of grid search for all parameters.

### Model testing

We tested the model performance against temporally and spatially independent PBD data. Temporally independent data consisted of the PBD observations from 1951 to 1970 at the Tidal Basin. These past PBD were compared with the predicted PBD by the calibrated model using daily temperature data from two adjacent weather stations near the Tidal Basin for both cultivars: 1) the Reagan National Airport weather station and 2) the Dulles International Airport weather station. The Dulles International Airport is located in the vicinity of Washington, DC (36.4 km from the Tidal Basin).

Spatially independent, recent PBD observations were obtained from the Project BudBurst Database and at the University of Washington (UW) campus in Seattle, WA. Four observations (Comer, GA, Germantown, TN, Bloomington, IN, for Yoshino and Charlotte, NC for Kwanzan) were mined from the Project BudBurst Database which compiles the phenological records collected by participating citizen scientists (pers. comm., Dennis Ward). In addition, we estimated recent PBD at UW from the campus newspapers and other mass-media including web search as well as our own observations for nine years of data between 1994 and 2011. Both Yoshino and Kwanzan cherry trees are grown in UW Seattle campus. We acquired temperature data for these locations from the corresponding National Oceanic and Atmospheric Administration (NOAA) climatological observations sites (e.g., Sea-Tac Airport in Seattle). The average distance between a PBD observation site and the corresponding climatological observation site was 38 km; all weather stations were located within 16 km with an exception of Bloomington, IN where the distance was 150 km. We calculated root mean square error (RMSE), bias, *r* ^2^, and mean absolute error (MAE) between the observed and the predicted PBD to evaluate model performance. The formula for these statistics can be found in [18], [29].

### Predicting the future PBD in the Mid-Atlantic region

We applied the model to forecast future cherry PBD throughout the Mid-Atlantic region surrounding the Tidal Basin including the states of Maryland, Virginia, and West Virginia in response to climate change. This regional forecast was made in 30-year intervals to follow the normal year method of the World Meteorology Organization [30]. The projected normals (averages over a prescribed 30-year interval) of monthly climate data between 2010 and 2100 were obtained from Consultative Group on International Agricultural Research (CGIAR)'s Research Program on Climate Change, Agriculture and Food Security (CCAFS) climate data archive (http://ccafs-climate.org). The datasets used in the study represent statistically downscaled regional projections based on the IPCC SRES scenario A1B and A2 [7], [31], [32]. We used regional climate projections derived from two general circulation models (GCM): ECHAM5 by Max-Planck Institute (MPI) for A1B scenario and CGCM2 by Canadian Center for Climate Modeling and Analysis (CCCMA) for A2 scenario. More details on these models are provided by Roeckner et al. [33] for MPI-ECHAM5 and by Flato and Boer [34] for CCCMA-CGCM2. We performed temporal downscaling of the monthly data into daily temperature data needed to run the phenology model by applying the harmonic analysis detailed in Chung et al. [16] and Seino [35]. The spatially and temporally downscaled datasets include daily maximum and minimum temperatures at a resolution of 30 arc-seconds (ca. 90 m) for the Washington, DC and surrounding areas including parts of Maryland, Virginia, and West Virginia. Using these datasets, we forecasted the future PBD of Yoshino and Kwanzan trees for three 30 normal year periods: the 2020 s (2010–2039), 2050 s (2040–2069), and 2080 s (2070–2099) at 30 arc-seconds spatial resolution.

## Results

### Model parameterization

**Figure 2:**
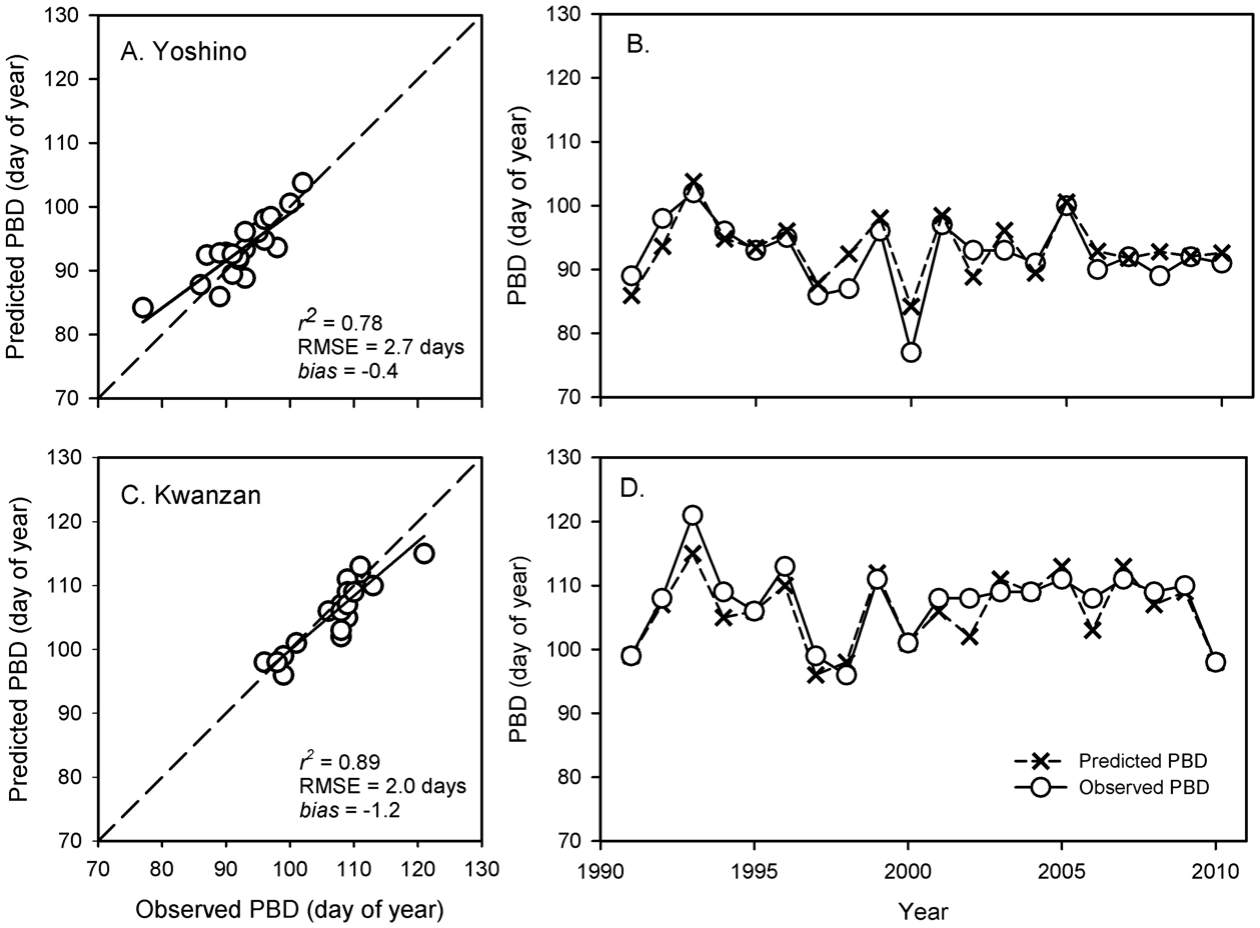
Model parameterization results for predicting peak bloom dates (PBD) of Yoshino (A, B) and Kwanzan cherry trees (C, D) in the Tidal Basin, Washington, DC during 1991–2010. Predicted PBD vs. observed PBD are shown in A (Yoshino) and C (Kwanzan). The temporal trends of PBD for the same period are shown in B (Yoshino) and D (Kwanzan). The observed PBD are represented by open cycles (○) with a solid line and the predicted PBD by cross symbol (×) with a dashed line.

The parameter optimization process yielded a set of parameter estimates that produced minimum RMSE between predicted and observed PBD during the 1991–2010 period in the Tidal Basin (Table 1). The estimates for both Yoshino and Kwanzan cherry trees are considerably different from the values estimated for *P. serrulata* var. *spontanea* by Jung et al. [19] (Table 1). In addition, the two-step model used in this study was capable of explaining most of the variability in PBD accurately for Yoshino (*r* ^2^ = 0.78, MAE = 2.4 days, RMSE = 2.7 days, bias = 0.9) and Kwanzan (*r* ^2^ = 0.89, MAE = 2.1 days, RMSE = 2.0 days, bias = −1.2) cherry trees in the Tidal Basin over the period of 1991 through 2010 (Fig. 2).

**Table 1:** Parameter estimates for the cherry cultivars used in the study.

Cultivar	Parameter estimates		
	*T_c_*	*R_c_*	*R_h_*
	(°C)	(chill days)	(anti-chill days)
*Prunus*×*yedoensis ‘*Yoshino’	4.3	−78.9	221.2
*Prunus serrulata* ‘Kwanzan’	5.3	−114.0	289.1
*Prunus serrulata* var. *spontanea*	7.0	−110.0	123.5

Also included for comparison are the estimates for (*Prunus serrulata* var. *spontanea*) in Korea from Jung et al., (2006). *T_c_* is represents the base threshold temperature, *R_c_* is the is the required chilling units, and *R_h_* is the required heating (anti-chill) units.

### Model testing

**Figure 3:**
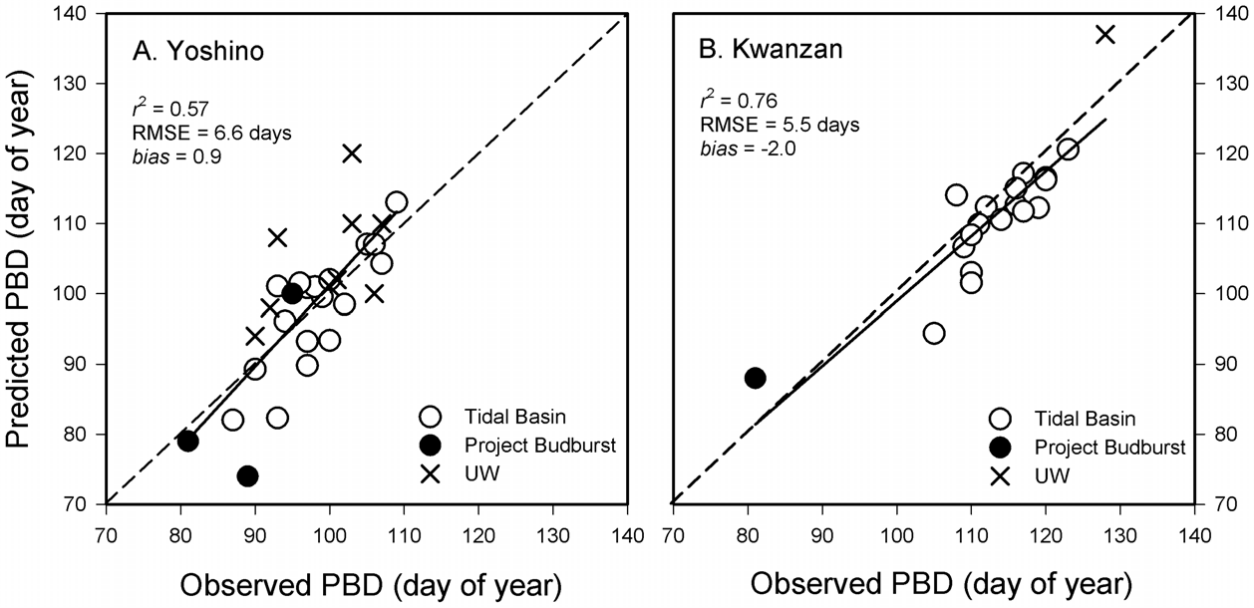
Model performance against independent PBD data sets for Yoshino (A) and Kwanzan (B). Open circles represent past PBD data from 1951 to 1970 at the Tidal Basin, Washington, DC. Closed circles are PBD data of Project Budburst for Comer, GA, Germantown, TN, Bloomington, IN, for Yoshino and Charlotte, NC for Kwanzan. Cross symbols are recent PBD data at the University of Washington, Seattle, WA between 1994 and 2001.

Applying the new parameter estimates for Yoshino and Kwanzan cherry trees, we tested the model performance against temporally and spatially independent data sets that included PBD observations from 1951 to 1970 at the Tidal Basin, from 1994 to 2011 at the University of Washington campus in Seattle, WA, and from the Project BudBurst database recorded in 2008 at the four locations described in Methods section. The model performed well to predict PBD of Yoshino (*r* ^2^ = 0.57, MAE = 5.1 days, RMSE = 6.6 days, bias = 0.9) and Kwanzan (*r* ^2^ = 0.76, MAE = 4.4 days, RMSE = 5.5 days, bias = −2.0) for the combined data (Fig 3). When tested against the past PBD data (1951–1970) from the Tidal Basin only, the model performance was further enhanced for Yoshino (*r* ^2^ = 0.64, MAE = 4.0 days, RMSE = 3.5 days, bias = −0.4) while it remained similar for Kwanzan (*r* ^2^ = 0.67, MAE = 3.9 days, RMSE = 3.0 days, bias = −3.2). The past PBD predictions at Tidal Basin using the temperature data from the Dulles International Airport resulted in similar performance (data not shown).

### Future changes in cherry peak bloom dates

**Figure 4:**
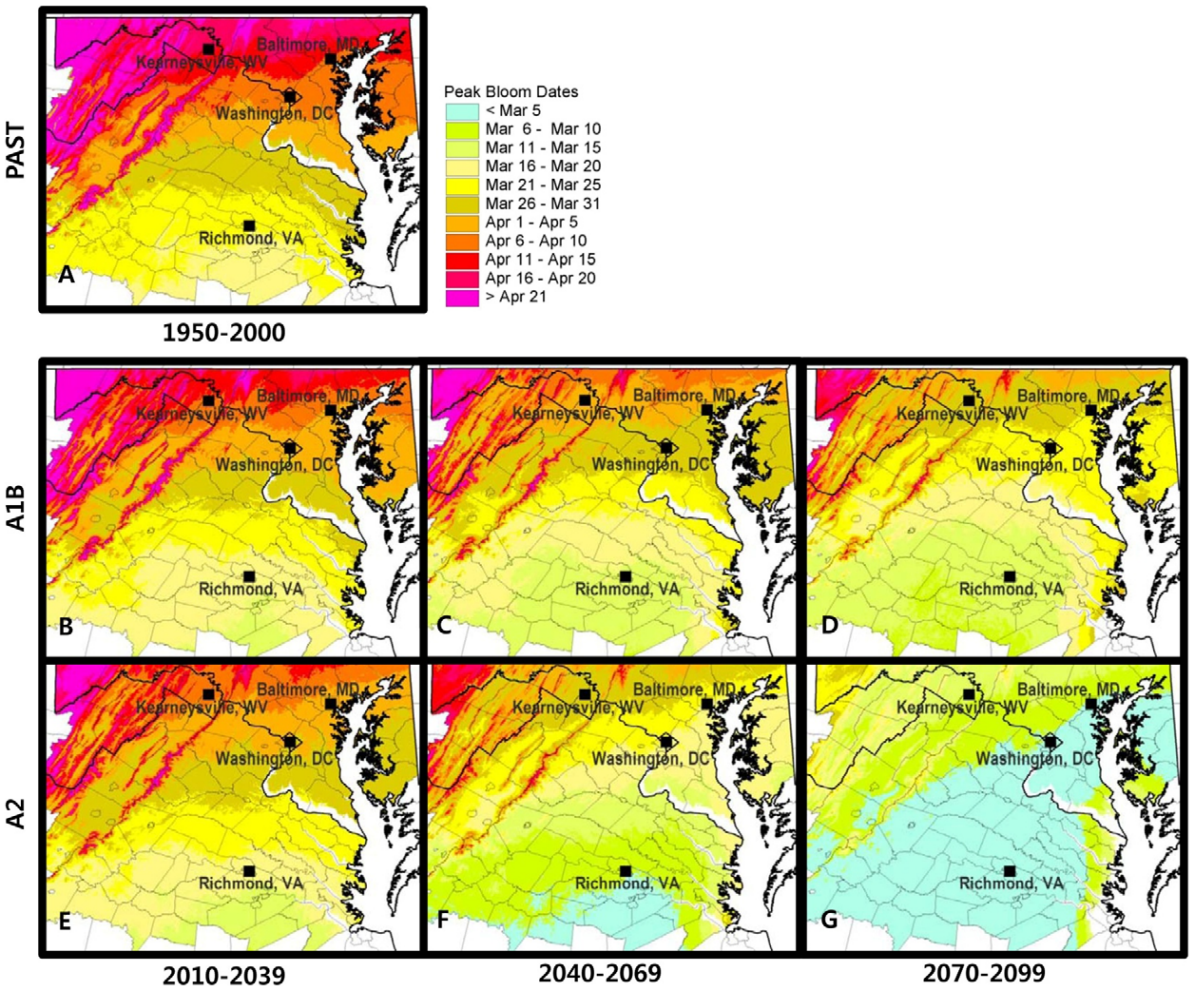
Past and projected peak bloom dates (PBD) of the Yoshino cherry trees in Washington, DC and surrounding area. The past PBD from 1950 to 2000 (A), and the projected PBD for the three climatological normal years 2010–2039 (B and E), 2040–2069 (C and F), and 2070–2100 (D and G) are shown at 5-days intervals. The future projections were made under the IPCC SRES A1B (middle panels) and A2 (bottom panels) scenarios based on MPI-ECHAM5 and CCCMA-CGCM2 general circulation models. Locations of the four cities in Table 2 are also shown.

Gridded daily maximum and minimum temperatures from the past (1950–2000) and the three projected climatological normals (2010–2039, the 2020 s; 2040–2069, the 2050 s; 2070–2099, the 2080 s) have been applied to the phenology model to create mean PBD predictions for Yoshino and Kwanzan cherry trees in the Tidal Basin and the surrounding region (Fig. 4). For Yoshino, the predicted mean PBD of the region in the past years ranged from March 14 in southern Virginia through May 18 in the Appalachian mountain area in West Virginia (Fig. 4A). For the 2020 s of A1B, the predicted mean PBD at the Tidal Basin was April 1 with the spatial variation in the surrounding region ranging from March 12 to May 13 (Fig. 4B). By the 2050 s, the model predicted that the mean PBD at the Tidal Basin is likely to be accelerated to March 18 under A2 scenario (Fig. 4F). The model predicted a more dramatic shift in PBD for the entire region in the 2080 s (Fig. 4D and 4G); the predicted mean PBD of Yoshino cherry trees at the Tidal Basin were further accelerated to February 27 in the 2080 s under the A2 scenario (Table 2). A similar pattern was also found for Kwanzan under both emission scenarios (Table 2).

**Table 2:** Predicted mean peak bloom dates of cherry trees (*Prunus*×*yedoensis* ‘Yoshino’ and *Prunus serrulata* ‘Kwanzan’) at selected Mid-Atlantic locations in response to the projected climate under the A1B and A2 emission scenarios of MPI-ECHAM5 and CCCMA-CGCM2 general circulation models, respectively, during 2010–2099.

Cultivar	City	A1B			A2			SD	
		2020 s	2050s	2080 s	2020 s	2050 s	2080 s	A1B	A2
*Prunus*×*yedoensis* ‘Yoshino’	Washington, DC	April 1	March 27	March 22	March 31	March 19	March 4	4.1	12.1
	Baltimore, MD	April 2	March 28	March 23	March 31	March 18	March 3	6.8	15.8
	Kearneysville, WV	April 11	April 4	March 30	April 7	March 29	March 11	5.1	11.8
	Richmond, VA	March 18	March 14	March 13	March 18	March 7	February 27	3.7	10.6
	SD	9.9	8.8	7.0	8.3	9.0	5.4		
									
*Prunus serrulata* ‘Kwanzan’	Washington, DC	April 17	April 11	April 6	April 13	April 3	March 17	4.6	12.7
	Baltimore, MD	April 18	April 12	April 7	April 14	April 3	March 17	4.9	16.6
	Kearneysville, WV	April 26	April 20	April 14	April 21	April 12	March 24	7.8	12.8
	Richmond, VA	April 4	March 30	March 28	April 2	March 24	March 12	4.6	11.4
	SD	9.1	8.7	7.0	7.9	7.8	4.9		

The observed mean peak bloom dates during 1971–2000 at the Tidal Basin are April 2 for Yoshino and April 15 for Kwanzan during a time period under each sc. SD represents one standard deviation across different time periods within a scenario at each location, or across the four locations.

In addition to examining the overall future PBD distribution, we extracted the predicted PBD of four specific locations in the region from the gridded results: Washington DC, Baltimore, MD, Kearneysville, WV, and Richmond, VA (Table 2). Our modeling results suggest that a considerable temporal variability is likely to emerge in the mean PBD between locations (see Table 2 for standard deviations between locations). Overall, the model predicted that by the 2080 s the mean PBD in these locations would take place approximately four weeks earlier than the current PBD under the A2 emission scenario (Table 2).

## Discussion

Genetically, Yoshino cherry is thought to be closely related to several varieties in *P. serrulata* including var. *spontanea* [21] but the origin of Yoshino cherry (*P*.×*yedoensis*) is unknown [20]. Phenologically, the three distinct sets of parameter estimates identified between our study and Jung et al. [19] suggest there are likely to be inherent differences in the physiology associated with dormancy release and thermal induction of flowering in these cultivars. The parameter estimates for the Yoshino cherry indicate that it is likely to be more sensitive to warmer temperatures during the spring than *spontanea* with a lower base temperature (*T*_c_), a lower chilling requirement (*R*_c_), and a greater heating (i.e., forcing; anti-chilling) requirement (*R*_h_). The early-flowering cherry cultivars have been thought to be more responsive to a changing climate than late-flowering cultivars, potentially affecting gene flow and pollination between genotypes [36]. In addition to the potential genotypic differences, different regional weather patterns could also create variable responses even for closely related cultivars. The influence of the Atlantic Ocean on the Mid-Atlantic States of the U.S. and that of the northern Pacific on Korea are likely to create different weather patterns. Such differences could alter the dormancy and flowering habits of the trees acclimated to each region even for identical cultivars. In our work, PBD are estimated to occur earlier in the coastal areas than in the inland areas; also expected is that the change in the mean PBD over time would be greater in the coastal areas compared to the inland. This is similar to the findings in South Korea, where the dormancy release of cherry trees in the southern coast was predicted to be more irregular compared to the inland because of a further increase in temperature along the coastal areas in the winter [37]. Overall, the model predictions suggest that dormancy release of these cultivars in the region may be substantially delayed whereas the floral development after dormancy release is accelerated in early spring by the end of the century; this may result in unpredictable blooming habits and abnormal floral development of the cherry trees.

It should be noted that an extended growing season and autumn warming in the previous year could delay the onset of autumn syndrome stages (e.g., growth cessation, leaf fall, dormancy induction) and subsequently affect the phenology of the following spring in temperate and boreal trees [10], [38], [39]. In the current model, we assumed that the onset of dormancy takes place on Oct 1 of the previous year without explicitly accounting for the effects of autumn climate on dormancy induction. It is uncertain if and to what degree the flowering cherries are sensitive to autumn temperatures and photoperiod prior to the onset of dormancy. The sensitivity to autumn climate could subsequently trigger a delay in dormancy release which could counter the advancement of spring phenological events such as budburst and flowering that are mostly driven by winter and early spring temperatures. An explicit implementation of autumn syndrome stages could improve the model's ability to predict spring phenology more mechanistically. However, this aspect remains to be a challenge in modeling temperate tree phenology [40]. Meanwhile, many observational studies relate recent warming trends to earlier spring phenology in the temperate region [6], [41], [42], [43]

We determined that the model performance was reasonable after testing it against the multiple independent datasets that had not been used for model calibration (Fig. 3). Ideally, testing the model against multiple long-term PBD data sets from multiple locations would have furthered our confidence in applying the model towards the future scenarios. To our best knowledge, after an extensive search for additional data sets across the U.S., we have learned that long-term, reliable PBD data availability is limited to the Tidal Basin in the Mid-Atlantic region. Nonetheless, additional recent PBD datasets from Seattle, WA and from Project BudBurst improved our confidence in applying the model towards the future for wider geographic areas. Overall, given the challenges in data availability, potential genetic discrepancies, and climatic differences between locations, the model has successfully accounted for the variability in PBD of Yoshino and Kwanzan cherry trees in the Tidal Basin and other locations. It produced promising performance results that allowed us to apply to the future scenarios and assess the impacts of climate change on tree phenology for an iconic species in the region.

The accuracy of phenology records depends strongly on the observer and the procedure used. Variability in the ecology and age of the trees being observed, microclimates surrounding the trees, and the subjectivity of the observer can all lead to errors in phenological records. Therefore, in order to improve the ability to test and apply the predictive phenology models, a standardized observation method of species-specific phenophases for institutions and scientists observing phenology are critical to create reliable, long-term data sets from multiple locations. As evidenced in our current study, research approaches to engage citizen scientists are likely to be an effective method for achieving this goal, provided well-defined phenophase standards are available for the species of interest (e.g., Project BudBurst, Floral Report Card Project, and USA National Phenology Network) [44], [45]. More independent phenological data on the budburst and leaf fall as well as the PBD of cherry trees for multiple locations and cultivars will greatly aid in improving the model and its usefulness for predicting the impacts of climate change on this temperate tree phenology.

Our results illustrate the utility of a simple but robust process-based thermal unit model as a tool for assessing the impacts of climate change on temperate tree phenology and for developing adaptation strategies in horticulture and forestry in response to a rapidly changing climate. For example, our results suggest that the timing of PBD and the window of the National Cherry Blossom Festival at the Tidal Basin may mismatch towards the second half of this century. This type of information can be useful in adaptive planning of this culturally and economically important event in the mid- to long-term perspectives. Furthermore, the model can also be useful for a diverse range of other applications such as planning and management of fruit crop production. For example, the model may be used to predict the flowering dates to schedule pollinators for apple, pear, peach trees, and other deciduous fruit trees [27]. In addition, this type of predictive model will become increasingly useful when it is capable of making real-time forecasts. However, the uncertainties involved in short-term weather forecasting (e.g., weeks, and months) present a bottleneck in applying the model for real-time predictions within the season. Therefore, an improved and novel modeling approach is called for to maximize the use of phenology models for making real-time decisions and in-season predictions.

In conclusion, we derived a new set of parameter estimates for the Yoshino and the Kwanzan cherry trees using the observed PBD data from 1991 to 2010 recorded at the Tidal Basin. We then tested the model performance against the past PBD data from 1951 to 1970 in Tidal Basin and from several other locations in more recent years. Model performance was satisfactory suggesting its applicability for predicting future flowering dates in response to climate change. We applied the model parameterized for Yoshino and Kwanzan cherries to predict the PBD throughout the 21^st^ century based on IPCC's A1B and A2 emission scenarios for the Tidal Basin and surrounding Mid-Atlantic region. Coupled with the regional climate projections, the model predicted considerable acceleration of PBD in both Yoshino and Kwanzan cherry trees in the region towards the end of the century. We anticipate that this type of simple but robust model based on known physiological processes would provide valuable insights for developing adaptation strategies to climate change in horticulture, conservation planning, restoration and other related disciplines and industries.

## References

[1] Menzel A, Fabian P (1999). Growing season extended in Europe.. Nature.

[2] Menzel A, Sparks TH, Estrella N, Koch E, Aasa A (2006). European phenological response to climate change matches the warming pattern.. Global Change Biology.

[3] Myneni RB, Nemani RR, Running SW (1997). Estimation of global leaf area index and absorbed par using radiative transfer models.. Ieee Transactions on Geoscience and Remote Sensing.

[4] Parmesan C, Yohe G (2003). A globally coherent fingerprint of climate change impacts across natural systems.. Nature.

[5] Richardson AD, Bailey AS, Denny EG, Martin CW, O'Keefe J (2006). Phenology of a northern hardwood forest canopy.. Global Change Biology.

[6] Abu-Asab MS, Peterson PM, Shetler SG, Orli SS (2001). Earlier plant flowering in spring as a response to global warming in the Washington, DC, area.. Biodiversity and Conservation.

[7] IPCC (2007). The Physical Science Basis.. Contribution of Working Group I to the Fourth Assessment Report of the Intergovernmental Panel on Climate Change.

[8] Chuine I, Beaubien EG (2001). Phenology is a major determinant of tree species range.. Ecology Letters.

[9] Cannell MGR, Murray MB, Sheppard LJ (1985). Frost avoidance by selection for late budburst in Picea-sitchensis.. Journal of Applied Ecology.

[10] Heide OM, Prestrud AK (2005). Low temperature, but not photoperiod, controls growth cessation and dormancy induction and release in apple and pear.. Tree Physiology.

[11] Heide OM (2008). Interaction of photoperiod and temperature in the control of growth and dormancy of Prunus species.. Scientia Horticulturae.

[12] Miller-Rushing AJ, Katsuki T, Primack RB, Ishii Y, Lee SD (2007). Impact of global warming on a group of related species and their hybrids: cherry tree (Rosaceae) flowering at Mt. Takao, Japan.. American Journal of Botany.

[13] McClellan A (2005). The Cherry Blossom Festival: Sakura Celebration: Bunker Hill Publishing, Boston.

[14] Jefferson RM, Fusonie AM (1977). The Japanese flowering cherry trees of Washington, D.C.: a living symbol of friendship.. US Department of Agriculture, Washington DC.

[15] Rutishauser T (2003). Cherry tree phenology..

[16] Chung U, Jung JE, Seo HC, Yun JI (2009). Using urban effect corrected temperature data and a tree phenology model to project geographical shift of cherry flowering date in South Korea.. Climatic Change.

[17] Primack RB, Higuchi H, Miller-Rushing AJ (2009). The impact of climate change on cherry trees and other species in Japan.. Biological Conservation.

[18] Cesaraccio C, Spano D, Snyder RL, Duce P (2004). Chilling and forcing model to predict bud-burst of crop and forest species.. Agricultural and Forest Meteorology.

[19] Jung JE, Kwon EY, Chung U, YJ I (2005). Predicting cherry flowering date using a plant phenology model.. Korean Journal of Agricultural and Forest Meteorology.

[20] Pooler MR (1999). Preservation and DNA fingerprinting of the historic Tidal Basin cherries.. Journal of Environmental Horticulture.

[21] Roh MS, Cheong EJ, Choi IY, Joung YH (2007). Characterization of wild Prunus yedoensis analyzed by inter-simple sequence repeat and chloroplast DNA.. Scientia Horticulturae.

[22] Seeley SD (1996). Modelling climatic regulation of bud dormancy;. Lang GA, editor: CAB International.

[23] Samish RM (1954). Dormancy in woody plants.. Annual Review of Plant Physiology and Plant Molecular Biology.

[24] Saure MC (1985). Dormancy release in deciduous fruit trees..

[25] Oh SD (2004). Fruit tree physiology in relation to temperature: Dormancy: Kilmogum Publication..

[26] Chuine I (2000). A unified model for budburst of trees.. Journal of Theoretical Biology.

[27] Zavalloni C, Andresen JA, Flore JA (2006). Phenological models of flower bud stages and fruit growth of ‘Montmorency’ sour cherry based on growing degree-day accumulation.. Journal of the American Society for Horticultural Science.

[28] Linkosalo T, Hakkinen R, Hanninen H (2006). Models of the spring phenology of boreal and temperate trees: is there something missing?. Tree Physiology.

[29] Retta A, Vanderlip RL, Higgins RA, Moshier LJ, Feyerherm AM (1991). Suitability of corn growth models for incorporation of weed and insect stresses.. Agronomy Journal.

[30] WMO (1989). Calculation of Monthly and Annual 30-Year Standard Normals, WCDP-No. 10, WMO-TD/No. 341..

[31] Hijmans RJ, Cameron SE, Parra JL, Jones PG, Jarvis A (2005). Very high resolution interpolated climate surfaces for global land areas.. International Journal of Climatology.

[32] Ramirez J, Jarvis A (2010). Downscaling global circulation model outputs: The Delta method..

[33] Roeckner E, Brokopf R, Esch M, Giorgetta M, Hagemann S (2006). Sensitivity of simulated climate to horizontal and vertical resolution in the ECHAM5 atmosphere model.. Journal of Climate.

[34] Flato GM, Boer GJ (2001). Warming asymmetry in climate change simulations.. Geophysical Research Letters.

[35] Seino H (1993). An estimation of distribution of meteorological elements using GIS and AMeDAS data.. Journal of Agricultural Meteorology.

[36] Miller-Rushing AJ, Katsuki T, Primack RB, Ishii Y, Lee SD (2007). Impacts of global warming on a group of related species and their hybrids: cherry tree (Rosaceae) flowering at Mt. Takao, Japan.. American Journal of Botany.

[37] Yun JI (2006). Climate change impact on the flowering season of Japanese Cherry (*Prunus serrulata* var.. spontanea) in Korea during 1941–2100. Korean Journal of Agricultural and Forest Meorology.

[38] Heide OM (2003). High autumn temperature delays spring bud burst in boreal trees, counterbalancing the effect of climatic warming.. Tree Physiology.

[39] Granhus A, Floistad IS, Sogaard G (2009). Bud burst timing in Picea abies seedlings as affected by temperature during dormancy induction and mild spells during chilling.. Tree Physiology.

[40] Vitasse Y, Francois C, Delpierre N, Dufrene E, Kremer A (2011). Assessing the effects of climate change on the phenology of European temperate trees.. Agricultural and Forest Meteorology.

[41] Thompson R, Clark RM (2008). Is spring starting earlier?. Holocene.

[42] Schwartz MD, Ahas R, Aasa A (2006). Onset of spring starting earlier across the Northern Hemisphere.. Global Change Biology.

[43] Chmielewski FM, Rotzer T (2001). Response of tree phenology to climate change across Europe.. Agricultural and Forest Meteorology.

[44] Havens K, Schwarz J, Vitt P Using phenology to engage public audiences in climate change issues;.

[45] Henebry GM, Betancourt JL (2010). Toward a U.S. national phenological assessment.. Eos.

